# The immunity and redox clocks in mice, markers of lifespan

**DOI:** 10.1038/s41598-024-51978-9

**Published:** 2024-01-19

**Authors:** Judith Félix, Irene Martínez de Toda, Estefanía Díaz-Del Cerro, Fernando Gil-Agudo, Mónica De la Fuente

**Affiliations:** 1 Department of Genetics, Physiology and Microbiology (Animal Physiology Unit), Faculty of Biological Sciences, Complutense University of Madrid, 28040 Madrid, Spain; 2 Instituto de Investigación Sanitaria Hospital, 12 de Octubre (imas12), 28041 Madrid, Spain

**Keywords:** Immunology, Biomarkers

## Abstract

Immune function and redox markers are used for estimating the aging rate, namely biological age (BA). However, it is unknown if this BA and its changes can be reflected in longevity. Thus, we must quantify BA in experimental animals. In peritoneal immune cells of 202 female mice (ICR/CD1) in different ages, 10 immune and 6 redox parameters were evaluated to construct two mathematical models for BA quantification in mice by multiple linear regression. Immune and redox parameters were selected as independent variables and chronological age as dependent, developing two models: the Immunity and the Redox Clocks, reaching both an adjusted R^2^ of 80.9% and a standard error of 6.38 and 8.57 weeks, respectively. Both models were validated in a different group of healthy mice obtaining a Pearson’s correlation coefficient of 0.844 and 0.800 (*p* < 0.001) between chronological and BA. Furthermore, they were applied to adult prematurely aging mice, which showed a higher BA than non-prematurely aging mice. Moreover, after positive and negative lifestyle interventions, mice showed a lower and higher BA, respectively, than their age-matched controls. In conclusion, the Immunity and Redox Clocks allow BA quantification in mice and both the ImmunolAge and RedoxAge in mice relate to lifespan.

## Introduction

The aging process is defined as the progressive and generalized deterioration of the functions of the organism that leads to a reduced capacity to adaptively react to changes and preserve homeostasis^1^. We must consider that the functional decay that defines the aging process, and which begins when adulthood is reached, does not occur at the same rate in all subjects and, therefore, not all individuals in a population with the same chronological age (CA) are aging at the same rate. Based on this fact, the concept of “Biological Age” (BA) arises, which aims to estimate the rate at which each individual is aging and, therefore, is more indicative than CA of how they are aging and their lifespan^1–3^, being much more useful for assessing the overall health state of the individual than CA^4^.

One of the problems associated with BA is its quantification, which requires the determination and validation of a series of biomarkers of aging^5^. Aging is associated with many changes at all levels of biological organization (molecular, cellular, and physiological). Therefore, several parameters have been proposed as markers of BA. These include physiological (respiratory function, systolic blood pressure, hematocrit, etc.)^6,7^, biochemical (albumin, cholesterol, urea)^8,9^, genetic (telomeric length, circulating DNA fragments)^10,11^ and epigenetic markers (based on the degree of methylation of CpG islands^12^) that have led to the development of different models to estimate BA such as the telomere length or the epigenetic clock, among others^12,13^.

The function of the immune system has also been proposed as an excellent marker of health and the rate at which we age^14–16^. It is known that the immune system deteriorates with age, a process that is known as immunosenescence, being responsible for age-related increased morbidity and mortality. In fact, according to the oxidative-inflammatory theory of aging, which brings together concepts from Harman's oxidation theory^17^ and the term *Inflammaging* proposed by Franceschi^18^, the immune system is not only a passive marker of how we age but also a modulator of the aging process^1,14^. Based on this premise, we developed a mathematical model for BA prediction in humans based on immune parameters, “the Immunity Clock”^19^. This model includes five markers of immune cell function: the cytotoxic natural killer activity against tumor cells, the phagocytic and chemotactic capacity of neutrophils, and the chemotactic and proliferative capacity of lymphocytes. The mathematical model was constructed by multiple linear regression (MLR) achieving an R^2^ adjustment of 80.3% and a standard error of 4.74 years in the quantification of BA.

Moreover, the oxidative-inflammatory theory of aging states that what underlies the age-related functional decline of immune cells, and all cells of the organism, is the establishment of a chronic oxidative stress^1,5,14,20^. In support of this idea, several studies have shown an age-related increase of oxidative stress parameters in different cells and tissues^21–24^. Given the basis of oxidative stress in the aging process, a series of oxidative and antioxidant parameters in peripheral blood in humans and mouse peritoneal leukocytes have also been proposed as possible markers of BA^5,20^. Among them, the antioxidant activities catalase (Cat), glutathione peroxidase (GPx), glutathione reductase (GR), and the concentration of oxidized (GSSG) and reduced (GSH) glutathione, and thiobarbituric acid reacting substances (TBARs) were proposed. Through principal component analysis, it was found that different age groups in humans had a characteristic redox signature based on both oxidant and antioxidant parameters, what we have called the Human Redox Signature of aging^20^.

BA quantification based on immune and redox parameters in humans has been related to both successful and premature aging^19,20^. Nevertheless, the link between BA and lifespan cannot be tested in humans due to the long lifespan of our species. Thus, there is a need to develop mathematical models for BA assessment in mice to be able to ascertain if changes in BA do translate into a differential lifespan.

Therefore, this work aims to develop two mathematical models by multiple linear regression (MLR) for BA quantification in mice, one based on immune markers and another one based on redox parameters. The models will be validated in healthy mice, in mice with and without premature aging, and in mice after different positive and negative lifestyle interventions. Moreover, the relationship between BA quantified by these models and lifespan will also be explored.

## Results

### Immunity clock model

To develop the mathematical models for BA quantification a stepwise forward methodology was followed, which first selects the variable that most explains the dependent variable, then the next one, and so on. The criteria for variable inclusion into the model are that they had a *p*<0.05.

The multiple linear regression (MLR) analysis was performed using CA as the dependent variable and 10 parameters of immune function assessed in peritoneal leukocytes from 202 mice as independent variables: macrophage phagocytic index and efficacy, macrophage and lymphocyte chemotaxis, Natural Killer activity, basal lymphoproliferation and in response to the mitogens concanavalin A and lipopolysaccharide, and percentage of stimulation with concanavalin A and lipopolysaccharide. Table 1 shows the different steps of model construction.

**Table 1:** Immunity Clock model construction through the stepwise forward method from peritoneal leukocytes of 202 mice.

	Step 1	Step 2	Step 3	Step 4
Constant (β_0_)	83.799 (1.793)	89.263 (1.737)	95.493 (2.315)	105.222 (2.227)
Lymphoproliferation Concanavalin A (β_1_)	− 0.029*** (0.003)	− 0.018*** (0.003)	− 0.014*** (0.003)	− 0.012*** (0.003)
Phagocytic Index (β_2_)		− 0.020*** (0.003)	− 0.019*** (0.003)	− 0.018***(0.003)
Natural Killer Activity (β_3_)			− 0.238*** (0.064)	− 0.212*** (0.062)
Lymphocyte chemotaxis (β_4_)				− 0.010** (0.004)
R^2^	63.8%	87.2%	89.6%	90.6%
Adjusted R^2^	63.3%	75.4%	79.3%	80.9%

Each value shows the estimated coefficient and the standard error for each coefficient is shown in brackets. **p < 0.01, ***p < 0.001. No standardized beta coefficients (β) represent the amount of change in a dependent variable due to a 1-unit change in the independent variable. R^2^ is a statistical measure of how well the regression predictions approximate the real data points. An R^2^ of 100% indicates that the regression predictions perfectly fit the data.

**Figure 1:**
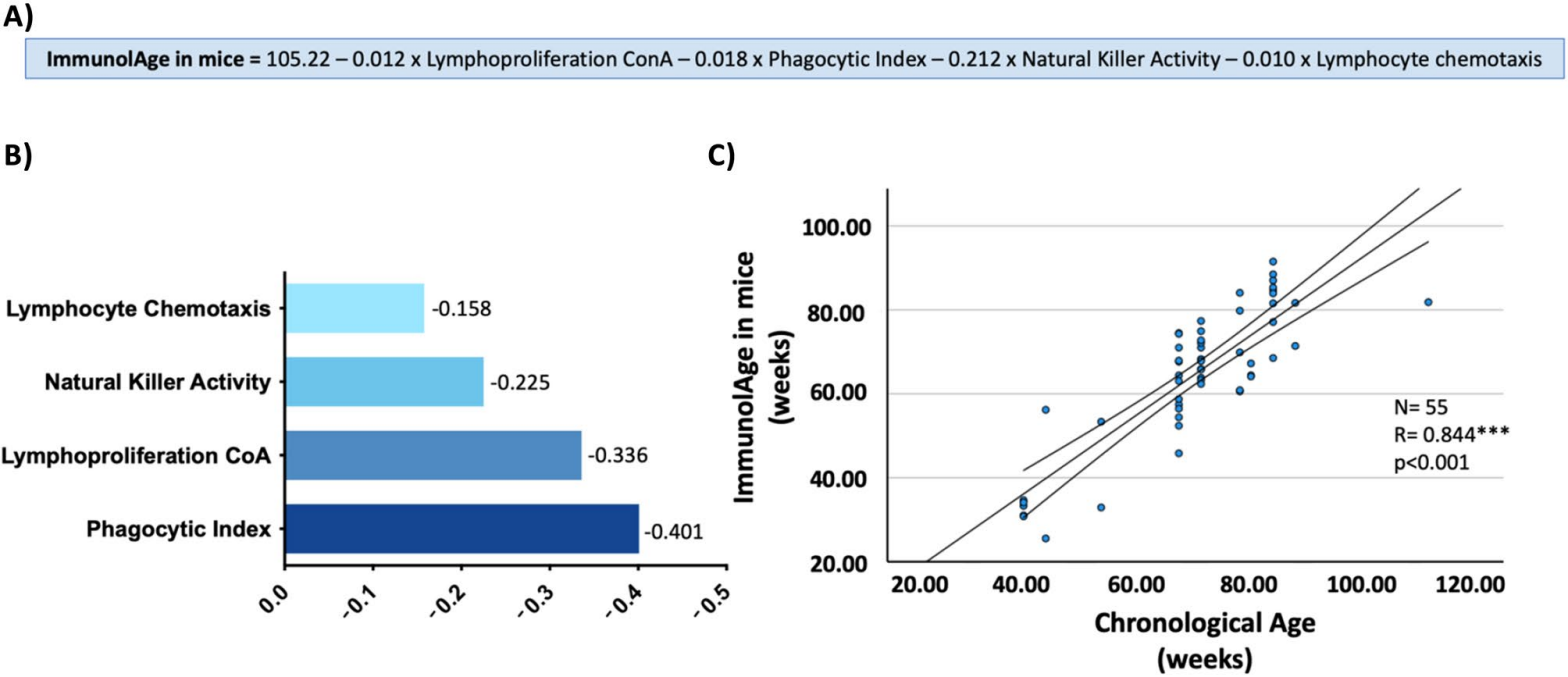
(**A**) Immunity Clock formula for the estimation of the ImmunolAge in mice. (**B**) Standardized beta coefficients for each immune function. (**C**) Chronological age vs ImmunolAge in a different set of healthy mice. Pearson’s correlation coefficient between chronological age and ImmunolAge in healthy mice is 0.844 (*p* < 0.001).

In the first step, lymphoproliferation with concanavalin was selected, then the phagocytic index, thirdly, Natural Killer activity, and, finally, lymphocyte chemotaxis. The rest of the variables were not included in the model as they did not fulfill the inclusion criteria. With the inclusion of the four variables, the model reached an adjusted R^2^ of 80.9% and a standard error of 6.38 weeks for BA estimation. The generated mathematical equation can be seen in Fig. 1A. We will refer to the BA predicted by the Immunity Clock model as ImmunolAge in mice.

To know which variable has the greatest effect on the mathematical model, we performed a standardization of the beta coefficients of each variable. In our case, the phagocytic index has the greatest weight, followed by lymphoproliferation with concanavalin A, Natural Killer activity, and lymphocyte chemotaxis (Fig. 1B).

To validate the model, another group of healthy mice (N = 55) that were not used for the construction of the model was used. After the application of the model in these animals, a Pearson’s correlation coefficient of 0.844 (*p* < 0.001) was obtained between CA and ImmunolAge (Fig. 1C).

### Redox clock model

For the construction of the Redox Clock model, the same steps were followed as in the case of the Immunity Clock model.

In the MLR, CA was used as the dependent variable, while the independent variables were glutathione reductase activity (GR), glutathione peroxidase activity (GPx), the concentration of oxidized (GSSG) and reduced (GSH) glutathione, GSSG/GSH ratio and concentration of TBARs assessed in peritoneal leukocytes from 202 mice. Table 2 shows the steps of model construction.

**Table 2:** Redox clock model construction through the stepwise forward method from peritoneal leukocytes of 202 mice.

	Step 1	Step 2	Step 3	Step 4	Step 5	Step 6
Constant (β_0_)	63.177 (2.376)	70.274 (3.437)	68.951 (3.190)	66.872 (2.980)	60.128 (2.743)	55.375 (2.895)
GSSG/GSH (β_1_)	3.276*** (0.413)	2.300*** (0.394)	1.262** (0.386)	0.626 (0.378)		
Glutathione Reductase (GR) (β_2_)		− 1.100*** (0.212)	− 1.067*** (0.178)	− 0.642***(0.189)	− 0.663*** (0.192)	− 0.619** (0.188)
Oxidized Glutathione GSSG (β_3_)			964.451*** (187.109)	1150.679*** (172.636)	1313.151***(144.239)	1235.051*** (146.007)
Reduced Glutathione GSH (β_4_)				− 979.061*** (240.669)	− 1144.330***(222.294)	− 904.249*** (247.794)
Glutathione Peroxidase GPx (β_5_)						− 0.097* (0.048)
R^2^	50.8%	66.0%	76.6%	81.8%	80.9%	82.1%
Adjusted R^2^	50.0%	64.9%	75.4%	80.5%	79.9%	80.9%

Each value shows the estimated β coefficient and the standard error for each coefficient is shown in brackets. *p < 0.05, **p < 0.01, ***p < 0.001. Non-standardized beta coefficients (β) represent the amount of change in a dependent variable due to a 1-unit change in the independent variable. R^2^ is a statistical measure of how well the regression predictions approximate the real data points. An R^2^ of 100% indicates that the regression predictions perfectly fit the data.

**Figure 2:**
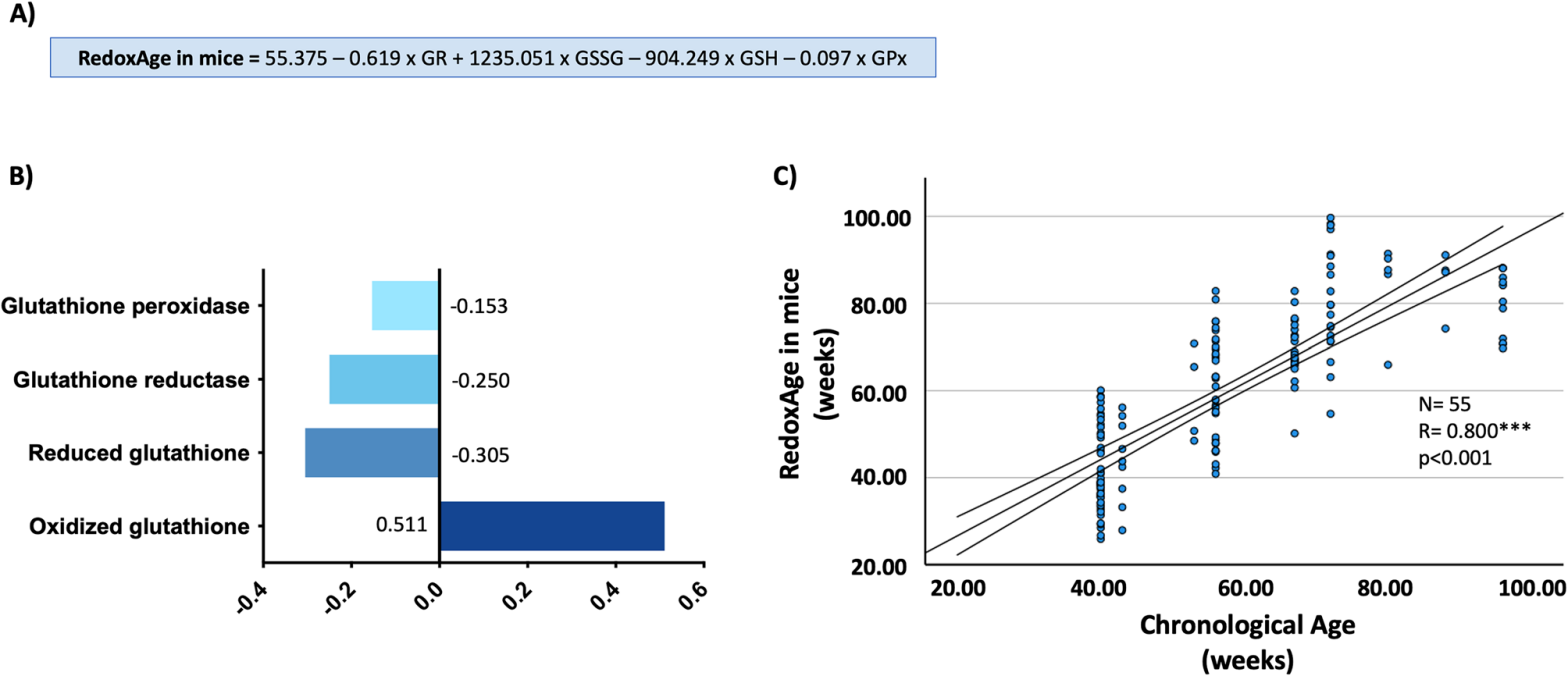
(**A**) Redox Clock formula for the estimation of the RedoxAge in mice. (**B**) Standardized beta coefficients for each redox parameter. (**C**) Chronological age vs RedoxAge in a different set of healthy mice. Pearson’s correlation coefficient between chronological age and RedoxAge in healthy mice is 0.800 (*p* < 0.001). GR, Glutathione Reductase; GSSG, Oxidized glutathione; GSH, Reduced glutathione; GPx, Glutathione Peroxidase.

In the first step, it selected the GSSG/GSH ratio, in the second the glutathione reductase activity, in the third the concentration of oxidized glutathione, in the fourth the concentration of reduced glutathione, in the fifth, it eliminated the GSSG/GSH ratio as a variable and, finally, it introduced glutathione peroxidase activity as the last variable. With these variables, the model presented an adjusted R^2^ of 80.9% and a standard error of 8.57 weeks. The generated mathematical equation can be seen in Fig. 2A. We will refer to the BA predicted by the Redox Clock as RedoxAge in mice.

Standardization of the beta coefficients for each variable was performed. In this case, the oxidized glutathione has the greatest weight in the developed model, followed by reduced glutathione, glutathione reductase, and glutathione peroxidase (Fig. 2B).

To validate the model, another set of healthy mice (N = 55) that were not used in the construction of the model was used. After the application of the model in these animals, a Pearson’s correlation coefficient of 0.844 (*p*<0.001) was obtained between CA and RedoxAge (Fig. 2C).

Moreover, we wanted to investigate if the inclusion of both sets of parameters, the immune and redox variables into a single combined model would increase the accuracy of BA prediction. Nevertheless, we obtained a lower prediction accuracy than with the Immunity and Redox clocks. This model adjusted the estimation by 73.9% (R^2^) with a standard error of 8.6 weeks including mostly immune function parameters (lymphoproliferation with Concanavalin A (ConA), phagocytic index, Natural Killer (NK) activity) and only one redox parameter (reduced glutathione concentration (GSH)). The obtained model was = 97.81 − 0.007 x ConA lymphoproliferation − 0.019 x phagocytic index − 0.311 x NK activity − 127.744 x GSH. However, given the lower predictive power, we did not use this combined model for further validation and application in the prematurely aging mice and the lifestyle interventions.

### Application of the immune and redox clocks in a model of premature aging in mice

Subsequently, the two mathematical models were applied in a mouse model of premature aging at the adult age in which prematurely aging mice (PAM) have a shorter lifespan than non-prematurely aging mice (NPAM) and NPAM have a shorter lifespan than exceptionally non-prematurely aging mice (ENPAM)^20,25,26^ to check if the BA of these mice is different from their CA (40 ± 1 week) and relate it to their longevity. This model of premature aging is based on an inadequate stress response when animals are subjected to the T maze, and how the different groups of animals are identified is described in the Methods section.

**Figure 3:**
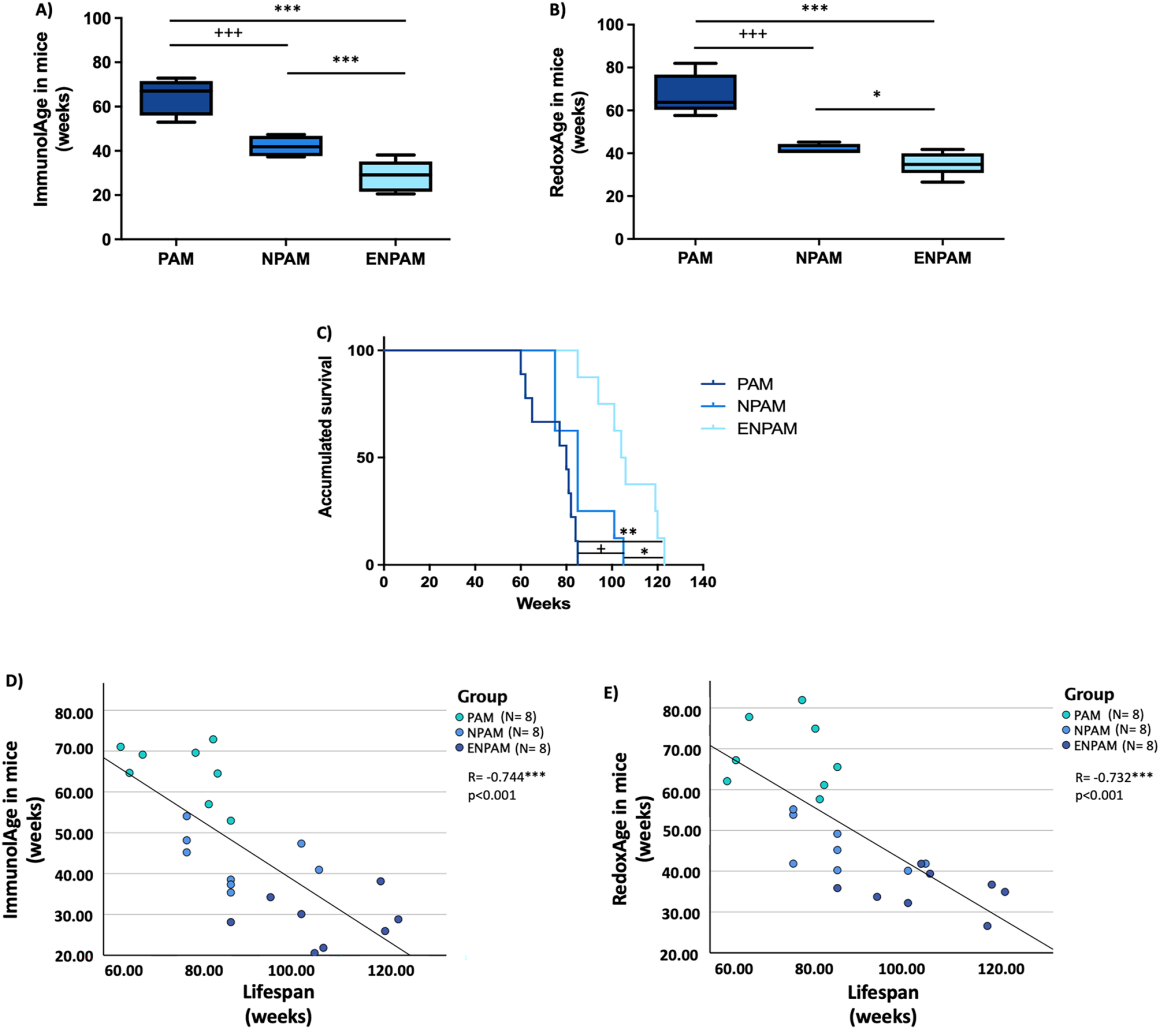
(**A**) Immunity Clock applied to prematurely aging and non-prematurely aging mice. (**B**) Redox Clock applied to prematurely aging and non-prematurely aging mice. (**C**) Kaplan‐Meier cumulative survival of PAM, NPAM, ENPAM. PAM: Prematurely aging mice, NPAM: non-prematurely aging mice, ENPAM: exceptional non-prematurely aging mice. *p < 0.05, **p < 0.01, ***p < 0.001 respect ENPAM.  + *p* < 0.05,  +  +  + *p* < 0.001 respect NPAM. (**D**) ImmunolAge vs lifespan in PAM, NPAM and ENPAM. Pearson’s correlation coefficient between ImmunolAge and lifespan is  − 0.744 (*p* < 0.001). (**E**) RedoxAge vs lifespan in PAM, NPAM, and ENPAM. Pearson’s correlation coefficient between RedoxAge and lifespan is  − 0.732 (*p* < 0.001).

When we applied the Immunity Clock (Fig. 3A), we observed that PAM display a higher ImmunolAge than NPAM and ENPAM (*p* < 0.001). NPAM show a similar ImmunolAge to their CA, while ENPAM exhibit a lower ImmunolAge than NPAM (*p *< 0.001).

With respect to the Redox Clock (Fig. 3B), PAM also display a higher RedoxAge than NPAM, and ENPAM (*p *< 0.001). NPAM exhibit a similar RedoxAge to their CA, while ENPAM show a lower RedoxAge than NPAM (*p *< 0.05).

Finally, when we look at the longevity of these groups of mice (Fig. 3C), we can observe that PAM have a shorter lifespan than NPAM and ENPAM (*p *< 0.05; *p *< 0.01, respectively) and that ENPAM have a higher lifespan than NPAM (*p *< 0.05). We also analyzed the existing relationship between the ImmunolAge and RedoxAge of each mouse and its final achieved lifespan (Fig. 3D,E). We found that both ImmunolAge and RedoxAge correlate negatively with lifespan. The correlation with ImmunolAge reached a Pearson’s correlation coefficient of  − 0.744 (Fig. 3D; *p *< 0.001), while with RedoxAge the obtained one was  − 0.732 (Fig. 3E; *p *< 0.001).

### Application of immunity and redox clocks on mice subjected to different lifestyle interventions

To further validate the applicability of the mathematical models developed, we subjected old and adult mice to different positive lifestyle interventions (such as nutritional or social) and young mice to a negative one, the ingestion of a sulfonated food additive (the full experimental design of these interventions is displayed in the Methods section).

**Figure 4:**
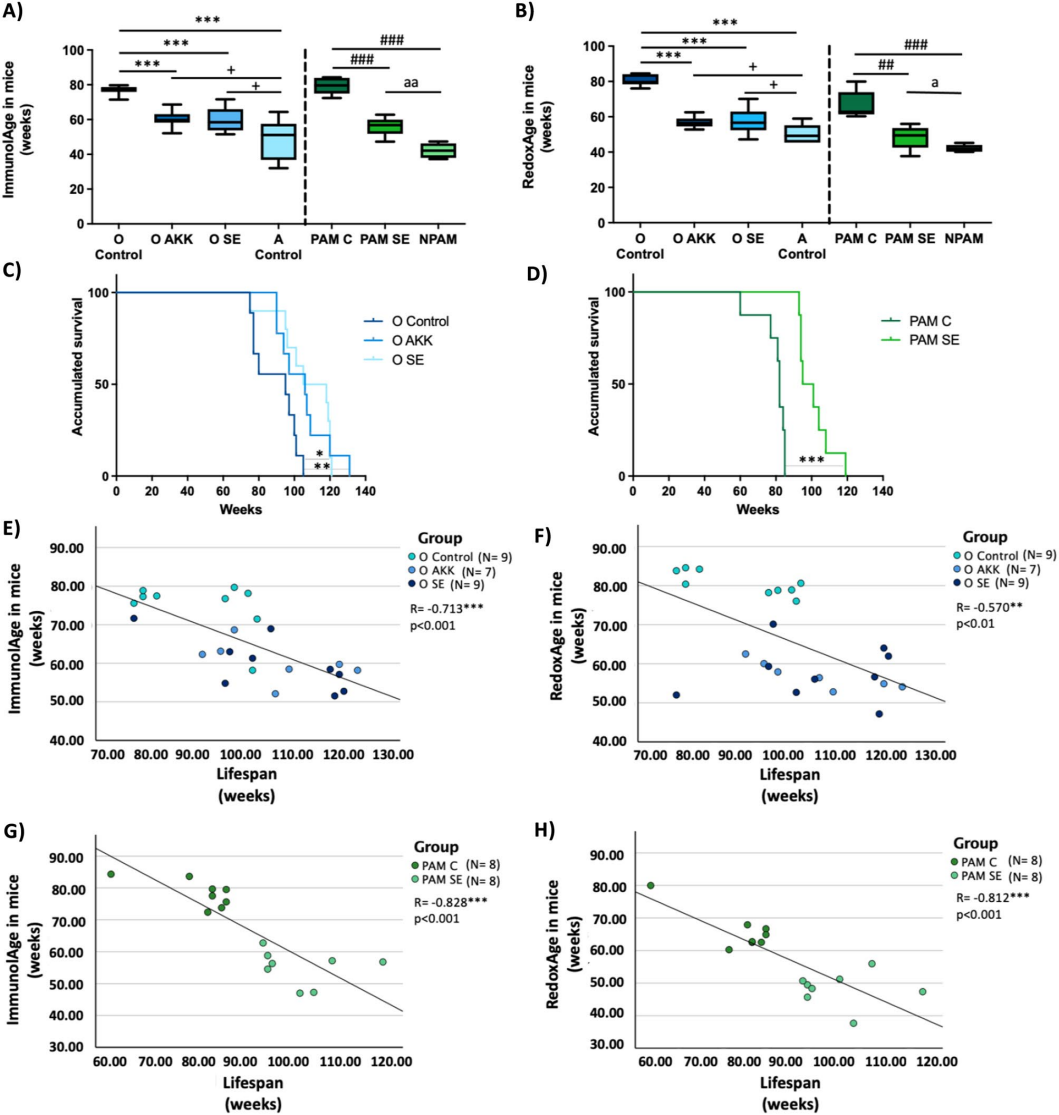
(**A**) Immunity Clock applied to mice after positive lifestyle interventions. (**B**) Redox Clock applied to mice after positive lifestyle interventions. (**C**) Kaplan Meier cumulative survival of O Control, O AKK and O SE. (**D**) Kaplan Meier cumulative survival of PAM C and PAM SE. (**E**) ImmunolAge vs lifespan in old mice after nutritional and social interventions. Pearson’s correlation coefficient between ImmunolAge and Longevity is  − 0.713 (*p* < 0.001). (**F**) RedoxAge vs lifespan in old mice after nutritional and social interventions. Pearson’s correlation coefficient between RedoxAge and lifespan is  − 0.570 (*p* < 0.01). (**G**) ImmunolAge vs lifespan in adult PAM after social interventions. Pearson’s correlation coefficient between ImmunolAge and lifespan is  − 0.828 (*p* < 0.001). (**H**) RedoxAge vs lifespan in PAM after social interventions. Pearson’s correlation coefficient between RedoxAge and Longevity is  − 0.812 (*p* < 0.001). O Control, Old control; O AKK, old mice after *Akkermansia* ingestion; O SE, old mice after cohabitation with adult mice; A Control, Adult control; PAM C, prematurely aging mice control; PAM SE, prematurely aging mice after cohabitation with exceptional non-prematurely aging mice. *p < 0.05, **p < 0.01, ***p < 0.001 respect O Control.  + *p* < 0.05 respect A Control. ##*p* < 0.01, ###*p* < 0.001 respect PAM C. a *p* < 0.05, aa *p* < 0.01 respect NPAM.

In the case of positive lifestyle interventions (Fig. 4A), when we applied the Immunity Clock, we observed that old mice after ingestion of the probiotic *Akkermansia mucciniphila* (O AKK), as well as old mice after cohabitation with adults (O SE), showed a lower ImmunolAge than old control mice (O Control) (*p* < 0.001) and still higher than that of adult control mice (A Control) (*p* < 0.05). Similarly, adult PAM after cohabitation with ENPAM (PAM SE) showed a lower ImmunolAge than the control PAM (PAM C) (*p* < 0.001), but higher than the ImmunolAge of NPAM (*p *< 0.001).

With respect to the Redox Clock (Fig. 4B), O AKK and O SE also display a lower RedoxAge than O Control (*p *< 0.001) and higher than adult control mice (*p *< 0.05). PAM SE showed a lower RedoxAge than PAM C (*p *< 0.01) and higher than that NPAM (*p *< 0.05).

Finally, when we look at the longevity of these groups of mice, we can observe that O AKK and O SE have a higher lifespan than the old control mice (Fig. 4C, *p *< 0.001; *p *< 0.05, respectively). PAM SE showed a higher lifespan than PAM C (Fig. 4D, *p *< 0.001). We also analyzed the existing relationship between individual ImmunolAge and RedoxAge of each mouse and its final achieved lifespan (Fig. 4E–H). We found that both ImmunolAge and RedoxAge correlate negatively with lifespan. In old mice, the correlation with ImmunolAge reaches a Pearson’s correlation coefficient of  − 0.713 (Fig. 4E; *p *< 0.001), while RedoxAge obtains one of  − 0.570 (Fig. 4F; *p *< 0.01). In PAM, the correlation with ImmunolAge reaches a Pearson’s correlation coefficient of  − 0.828 (Fig. 4G; *p *< 0.001), while RedoxAge obtains one of  − 0.812 (Fig. 4H; *p *< 0.001).

**Figure 5:**
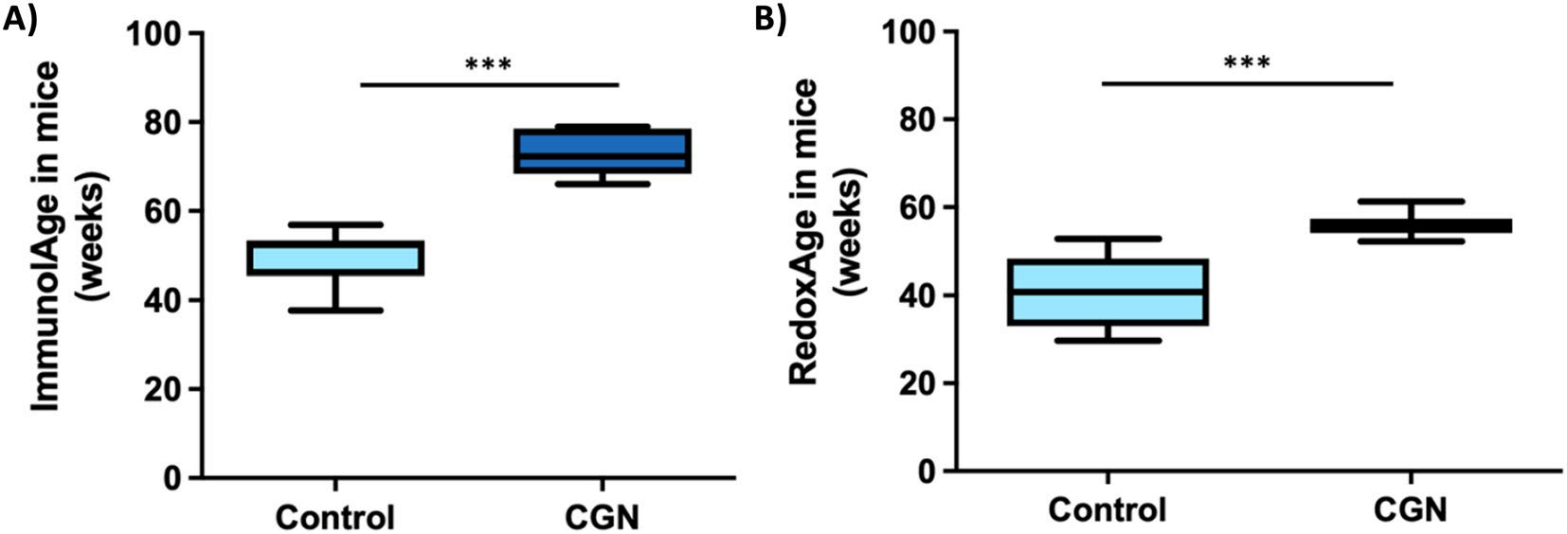
(**A**) Immunity Clock applied in mice after carrageenan ingestion in their first weeks of life. (**B**) Redox Clock applied in mice after carrageenan ingestion in their first weeks of life. CGN, adult mice after carrageenan ingestion; control, adult mice control. ***p < 0.001 respect control.

Concerning the impact on BA of a negative lifestyle intervention, the effects of the ingestion of carrageenan, a sulfonated food additive, in the first weeks of life were evaluated in healthy mice. These mice at adulthood (CGN) show both higher ImmunolAge (Fig. 5A; *p *< 0.001) and RedoxAge (Fig. 5B; *p *< 0.001) than controls.

## Discussion

Since the aging process is heterogeneous and each individual ages at a different rate, mathematical models for BA quantification are a very useful tool for research in the field of aging. First, they help identify those markers that are related to aging and longevity, which can be targeted to modulate the aging process and, secondly, they can be used to ascertain the effect that different interventions have on BA and, consequently, on lifespan, without the need to wait until the natural death of the individuals.

Nowadays several “Biological clocks” have been developed for BA quantification in humans^12,13^. These clocks use different parameters, such as the Immunity Clock developed by our research group^19^, the “Epigenetic Clock”^12^, or the “Telomeric Clock”^13^, among others. However, none of them can establish a direct relationship between BA and lifespan, due to the long lifespan of humans. The verification of this requires the development or the adaptation of such mathematical models to experimental animals, such as mice.

Therefore, this work is of utmost importance, since it has not only managed to recreate a mathematical model based on the immune parameters used in the Immunity Clock for humans to mice but also to develop another one based on redox markers in mice. The most important finding is that we demonstrate that ImmunolAge and RedoxAge in mice relate to their final achieved lifespan, independently of the age at which they are quantified.

In this study, we can first observe that the Immunity Clock in mice reaches an adjusted R^2^ value of 80.9% and a standard error of the estimation of 6.38 weeks with 4 variables: lymphoproliferation with concanavalin A, phagocytic index, Natural Killer activity, and lymphocyte chemotaxis. Given these results, we see that our model is not far from the Immunity Clock developed for humans, where an adjusted R^2^ of 80.3% and a standard error of 4.74 years were obtained^19^. However, the immune function variables included in the Immunity Clock for humans were different: the first variable included is Natural Killer activity, followed by lymphoproliferation with phytohemagglutinin, neutrophil chemotaxis, and neutrophil phagocytic index^19^. These differences could be due to the sample type used to obtain immune cells. The assessment of immune parameters in mice is performed using peritoneal leukocytes, whereas the determination in humans is performed in peripheral blood leukocytes^5,19^. In mice peritoneum, macrophages constitute the main cell type, finding a smaller number of lymphocytes and hardly any Natural Killer cells^27^. However, in human peripheral blood, we do not find circulating macrophages, as they are found as monocytes, whereas the main phagocytic cells are neutrophils. Meanwhile, there are many lymphocytes and Natural Killer cells^28^. Therefore, the cell type that predominates in each kind of sample could be related to the differences that appear between the mathematical models of the two species. “Nevertheless, despite these subpopulation differences, previous studies have shown that immune function studied in humans in peripheral blood and in mice in peritoneal leukocytes over the aging process follows a similar pattern^3^.

Taking into account that the age-related deterioration experienced by immune cells is associated with the establishment of chronic oxidative stress, due to the imbalance generated between oxidant and antioxidant compounds, in favor of the oxidants^1^, we developed another mathematical model in mice based on redox markers. These redox parameters would be easier to assess using commercial kits than those evaluated for immune function. The parameters to be assessed were chosen based on previous results^5,20^ where it was shown that different antioxidant defenses, such as glutathione reductase (GR) and peroxidase (GPx) activities, and reduced glutathione (GSH) concentration decrease with increasing age, while oxidative compounds such as oxidized glutathione (GSSG), as well as GSSG/GSH ratio or malondialdehyde (MDA) concentration increase with aging. The Redox Clock developed in mice, achieved an adjusted R^2^ of 80.9% with a standard error of 8.57 weeks. The model includes the following parameters: GR activity, GSSG, GSH, and GPx activity.

It is difficult to compare these results with previous studies since there are few focused on the determination of BA in mice using oxidative stress parameters. However, certain parameters selected in the Redox Clock, such as GSH concentration and GPx activity, have also been included in other mathematical models aimed at predicting mice lifespan in the adult age in the same strain of mice as the one used in this study^29^.

The BA prediction models developed in this study explain 80.9% of the variance of data and have a prediction error of 6.38 weeks for the Immunity Clock and 8.57 weeks for the Redox Clock. These results are not much different from those observed in other clocks developed in mice, such as the Epigenetic Clock, which explains 96% of the variance with an error of 7.11 weeks^30^. However, since our models are developed in peritoneal leukocytes, which can be isolated without sacrificing the animals, they allow us to monitor the animals until their natural death, so we can analyze the relationship between estimated BA and lifespan. Other models such as the Epigenetic Clock or the Telomere Clock are developed in blood samples or different tissues^30–32^, which implies the sacrifice of the animals and does not allow us to study the link with longevity. Moreover, some of the BA models developed in humans cannot be applied to mice, such as the Telomere Clock. Unlike the human telomerase (TERT), which is not expressed or expressed at extremely low levels in most human somatic tissues and cells, the mouse TERT expression is found in most adult tissues and organs. This difference likely results in, or at least contributes to, much longer telomeres (50–100 kb) in laboratory mice, in comparison to human telomeres (5–15 kb). As a result, telomere length is not apparently a limit to cellular lifespan in mouse cells^33^. Conversely, it has been demonstrated that the age-related changes in the immune and redox parameters analyzed in this study follow a similar pattern in humans and mice^3,20^. Given the age-related similarities, it seems plausible to think that changes in the ImmunolAge in humans would also have an impact on their lifespan.

To determine whether these models are capable of discriminating between animals of the same CA but different aging rates, we applied both the Immunity and the Redox Clock in adult mice with and without premature aging^25^. Thereby we found that PAM display a higher ImmunolAge and RedoxAge than NPAM and ENPAM and higher than their CA. Moreover, ENPAM show a lower ImmunolAge and RedoxAge than NPAM and their CA. Furthermore, when we studied the different lifespans of these groups of mice, we observed that PAM have a shorter lifespan than NPAM and ENPAM and that ENPAM live longer than NPAM, which is in agreement with previous studies^25^. Furthermore, it was observed that individual ImmunolAge and RedoxAge correlate with lifespan independently of the age at which they are quantified. Thus, it can be concluded that displaying a higher BA than the CA is related to lower longevity, while a lower BA is related to higher longevity also at the individual level.

In addition, many studies demonstrate how different changes in lifestyle (exercise, diet, probiotic intake, social environment...) have an impact on the health of humans and those performed on mice demonstrate that they can affect longevity^16,34,35^. Therefore, in the present study, we also wanted to evaluate if the Immunity and Redox Clocks can detect changes in BA in mice undergoing different lifestyle interventions. As positive interventions, we evaluated in old mice the effect of the probiotic *Akkermansia mucciniphila* intake for two weeks, and of the cohabitation for 15 min/day for 2 months with adult mice, two strategies that translate into a higher lifespan in mice^36,37^. We found, applying the Immunity and Redox Clocks, that old mice subjected to these interventions had a lower BA than controls of the same age and, consequently, lived longer. We also wanted to evaluate if our mathematical models were valid to detect differences in BA in prematurely aging adult mice subjected to the same intervention. For this purpose, we investigated the changes in BA in adult PAM after cohabitation with ENPAM 15 min/day for 2 months and found the same effect, lower ImmunolAge and RedoxAge, and longer longevity compared to the corresponding controls.

As a negative intervention, we studied the effect that ingestion in high amounts of a food thickener, carrageenan, in early life, could have at the adult age. Several studies have demonstrated the toxic effects of this additive as it has been observed to induce colitis, changes in lipid profile, immunotoxicity…^38–41^. We observed that mice that ingested carrageenan in early stages, upon reaching adulthood presented higher ImmunolAge and RedoxAge than controls of the same age. This suggests that these mice show premature aging in the adult age, and as occurs in other models of premature aging in mice^20,25,26^, probably their lifespan will be decreased. Unfortunately, we still do not have the lifespan data of these mice, as they are still alive. Nevertheless, these results show the importance of certain habits or decisions taken in the early stages of life, since these can have a lifelong impact on health and the rate of aging^42^.

However, although the results are important and allow us to establish a direct link between mice BA and lifespan, it should be taken into account that the development and validation of the Immunity and Redox Clocks have been carried out in ex-reproductive females of the ICR-CD1 strain, so further research is needed to ascertain if these models would apply to males of this strain, as well as to other strains of mice. In addition, the inclusion of pro-inflammatory markers in the development of these models, such as the TNF-α/IL − 10 ratio produced by immune cells^43^, could increase their predictive power.

In conclusion, we demonstrate that both the Immunity and Redox Clocks developed for BA quantification in mice not only are indicative of the rate at which each mouse is aging at a specific moment in its life, but that ImmunolAge and RedoxAge estimations strongly relate to the final achieved lifespan. Moreover, we demonstrate that both mathematical models can be used to identify adult mice displaying premature aging as well as to study the effect that different strategies or interventions have on the rate of aging in mice.

## Methods

### Animals

For this study, ex-reproductive female ICR-CD1 mice (Janvier, France) were used. We used the ICR-CD1 strain due to its higher genetic heterogeneity than that of inbred strains, which could facilitate the extrapolation of the results to humans^44^. Female mice were used for this study because male mice show aggressive and dominant behavior when caged together and it has been shown that social isolation causes alterations in the neuroimmunoendocrine communication affecting the aging rate^45^. We used ex-reproductive females given that it better replicates the most frequent situation in human aging. In addition, in ex-reproductive female mice, the cyclicity of the estrous cycle ceases (maintaining the diestrus phase), and because of that the hormones do not affect the parameters studied in the present work^46^.

They were maintained with *ad libitum* access to food and tap water under stable light-dark cycles (12/12h reversed light/dark cycle; lights off at 8:00 AM) to avoid circadian interferences. Temperature (22 ± 2ºC) and humidity (50–60%) were also controlled. The diet was under the recommendations of the American Institute of Nutrition for laboratory animals (A04 diet from Panlab S.L., Barcelona, Spain). Experiments were performed during the dark phase of the cycle (8:00–12:00h). The protocol was approved by the Experimental Animal Committee of Complutense University of Madrid (Spain) (PROEX 224.0/21). Animals were treated according to the guidelines of the European Community Council Directives ECC/566/2015 and ARRIVE guidelines.

One group of mice (N = 202) was used for immune function and redox data collection for the development of the mathematical models. For this purpose, peritoneal leukocytes were extracted at different stages of life until the moment of death. The ages at which samples were taken were 39, 40, 43, 53, 61, 67, 69, 71, 72, 78, 80, 84, 88, 96, 104, 112 and 120 weeks old. Several immunological parameters were analyzed in the peritoneal suspension: phagocytic index, phagocytic efficiency, macrophage chemotactic index, lymphocyte chemotactic index, Natural Killer activity, basal lymphoproliferative response, lymphoproliferative response to Concanavalin A (ConA), lymphoproliferative response to lipopolysaccharide (LPS), percentage of stimulation with ConA and percentage of stimulation with LPS to evaluate the state of the immune system. Different redox state parameters were also analyzed: glutathione reductase (GR) and peroxidase (GPx) activities, the concentration of oxidized (GSSG) and reduced (GSH) glutathione, and the concentration of thiobarbituric acid reactive substances (TBARs) to assess lipid peroxidation. Once the results of the different parameters were obtained in the different ages of the mice, a data matrix was generated to develop two mathematical models to calculate the BA of the mice.

To validate these models, another group of mice (N = 55) aged between 40 and 112 weeks was used, from which peritoneal leukocytes were extracted and the abovementioned immune functions and redox state parameters were assessed.

- Prematurely aging mice

To study the applicability of the models, another group of adult mice, all 40 weeks old, including mice with premature aging, was used^20,25,26^. This model is based on the selection of 40-week-old adult female mice of the ICR-CD1 strain that show a poor stress response when subjected to the T-maze test. For this, this test was performed once per week for a month, and the time that each mouse took to cross the intersection of the “T” with both hind legs was measured to distinguish PAM (prematurely aging mice, which needed more than 10 s to cross the intersection at each test the four times) from the NPAM (non-prematurely aging mice). Depending on the behavior of NPAM, they were divided into ENPAM (exceptional-non-prematurely aging mice; that required less than 10 s to cross the intersection at each test the four times) and NPAM (those that show an intermediate behavior spending less than 10 s to cross the intersection sometimes, and others more than 10 s). In this study, we included 8 PAM, 8 NPAM, and 8 ENPAM, housed in groups according to their rate of aging, i.e., in one cage there were only PAM, one had NPAM, and the other ENPAM. Finally, we waited until the natural death of the mice to study their longevity and its relation to the estimated BA.

- Lifestyle interventions

As positive interventions, we evaluated the effects in old mice (72 ± 1 weeks) of the ingestion of a probiotic, *Akkermansia mucciniphila*, for one month as well as of cohabitation for 2 months 15 min/day with adult mice (40 ± 1 weeks), as previously described^36,37^ sharing the control group for both interventions. Then, we evaluated the social environment intervention in PAM, this is, in adult mice with premature aging. For this, PAM cohabited 15 min/day for 2 months with ENPAM, as previously described^37^. We applied the mathematical models to 9 old control mice, 7 old mice that took the probiotic, 9 old mice after cohabitation, and 8 PAM control, and 8 PAM after cohabitation. For these interventions, we also waited until their natural death to study their longevity and establish a relationship with their BA.

As a negative intervention we evaluated the effects of high exposure to κ-carrageenan, a food thickener, for 2 weeks in mice during their first weeks of life (8 ± 1 weeks) and the impact this has on the adulthood of these mice (40 ± 1 weeks)^40^. 8 control adults and 8 mice taking carrageenan were used. In this case, longevity was not evaluated, since they are adult animals and their longevity is approximately two years, the animals are still alive.

### Extraction of peritoneal leukocytes

The extraction of peritoneal leukocytes was performed between 8:00 and 10:00 AM, to minimize circadian variations of the studied immune parameters following a protocol already described^3^. Without the use of anesthesia, mice were immobilized by taking a fold of the neck and the entire dorsal area of the animal, and the abdominal area was cleaned with 70% ethanol. Subsequently, 3 mL of sterile Hank's solution was injected intraperitoneally, and abdominal massage was performed. Thanks to this, 80% of the volume injected with the needle used for the injection of Hank's solution was recovered. The leukocytes of the peritoneal suspensions obtained were identified by their morphology (macrophages or lymphocytes) and quantified (number of cells/mL) in a Neubauer chamber using optical microscopy (x40). Cell viability was assessed by trypan blue exclusion test and only suspensions with a viability greater than 95% were used.

### Immune function parameters

- Macrophage and lymphocyte chemotaxis

It was performed following the technique described by Boyden (1962) and modified by De la Fuente's group^3^. It was evaluated by taking 300 μL of the peritoneal suspension containing macrophages or lymphocytes, adjusted to 500,000 cells/mL of Hank's solution that was deposited in the upper compartment of a Boyden chamber separated by a nitrocellulose filter with pores of 3 μm in diameter. In the lower compartment of the Boyden chamber 400 μL of formylated peptide (N-formyl-methionyl-leucyl-phenylalanine), a chemoattractant agent, was added to induce chemotaxis. After 3 hours of incubation at 37 °C and 5% CO_2_, filter-bound cells were fixed with 50% methanol and 75% ethanol and stained with azur-eosin (GIEMSA, PANREAC). Finally, the number of macrophages or lymphocytes that had passed through the filter (found on the underside) was counted by optical microscopy, and the chemotactic index (C.I.) was calculated.

- Macrophage phagocytosis

For this, the technique described by De la Fuente^3,47^ was used. 200 μL of peritoneal suspension adjusted to 500,000 macrophages/mL were incubated on migration inhibition factor (MIF) plates for 30 min. The adherent monolayer was washed with phosphate-buffered saline (PBS) at 37 °C and 20 μL of latex beads (1.09 μm diluted 1% PBS, Sigma-Aldrich) were added. After 30 min incubation, the sample was fixed with 50% methanol and stained with azur-eosin blue (GIEMSA, PANREAC). The number of particles per 100 macrophages (phagocytic index) and the percentage of macrophages that ingested at least one particle (phagocytic efficiency) were determined by optical microscopy (100x).

- Natural Killer activity

For this purpose, an enzymatic colorimetric kit (Cytotox 96 TM Promega, Boehringer Ingelheim, Germany) based on the determination of lactate dehydrogenase (LDH) released by cytolysis of target cells (tumor cells) was performed using tetrazolium salts. The peritoneal suspension (adjusted to 10^6^cells/mL culture medium) was added to 96-well U-bottom culture plates with target cells (murine YAC-1 tumor cells) in a 10:1 ratio. After 4 hours of incubation, LDH was measured by the addition of the enzyme-substrate at an absorbance of 490 nm.

The formula to calculate this function is:

$$\text {Lysis} \%=\frac{\text{Problem lysis}-\text{Effector cells spontaneous lysis}-\text{Tumor cells spontaneous lysis }} {\text {Tumor cells total lysis}- \text{Tumor cells spontaneous lysis}} x 100$$

where problem lysis of effector cells refers to the mean of well absorbances where the observed lysis is caused by the action of effector cells (peritoneal leukocytes) on target cells (YAC-1). Spontaneous lysis of effector cells is lysis due to the death of peritoneal leukocytes naturally during the process. The total lysis of target cells is the mean absorbance of those wells where all tumor cells have been lysed by the addition of a lysis solution. And finally, the spontaneous lysis of tumor cells is the mean of the lysis absorbances due to natural tumor cell death during the process^3^.

- Lymphoproliferative response

Peritoneal leukocyte suspensions adjusted to 10^6^ lymphocytes/mL of complete RPMI (supplemented with gentamicin (1 mg/mL) and 10% fetal bovine serum (Gibco), previously heat decomplemented for 30 min at 56 °C), were incorporated in aliquots of 200 μL/well, in 96-well plates. The following were added to the wells: 20 μL of RPMI medium in the case of the basal condition and 20 μL of Concanavalin A (ConA) or lipopolysaccharide (LPS) (1μg/mL) for the mitogen response. After 48 hours of incubation, tritiated thymidine was added, followed by another 24 hours of incubation. Cells were fixed on a filter by an automated machine and thymidine tritiated was measured in a microbeta counter. The results were expressed as counts per minute (c.p.m.) for basal and stimulated conditions and, in addition, the percentage of stimulation was assessed, that is, mitogen-stimulated lymphoproliferation divided by basal lymphoproliferation x100 into cases of mitogen-stimulated proliferative response^3^.

### Oxidative stress parameters

- Glutathione reductase activity (GR)

The glutathione reductase activity was analyzed following the methodology described^48^. Peritoneal leukocytes (10^6^ cells/mL) were resuspended in oxygen-free phosphate buffer (pH 7.4, 50 mM with 6.3 nM EDTA). They were then sonicated and, after centrifugation at 3200 g at 4 °C for 20 min, supernatants were collected. GSSG (80 mM) was used as substrate and, following the decrease in absorbance by oxidation of NADPH at 340 nm for 4 min, the activity was calculated. Results are expressed as milliunits (mU) of GR per milligram of protein (mU GR/mg protein).

- Glutathione peroxidase activity (GPx)

The glutathione peroxidase activity was assayed using a previously described method with some modifications^5^. Peritoneal leukocytes (10^6^ cells/mL) were resuspended in oxygen-free phosphate buffer (pH 7.4, 50 mM). They were then sonicated and, after centrifugation at 3200 g at 4 °C for 20 min, the supernatants were collected. Cumene hydroperoxide (cumene-OOH; Sigma) was used as substrate. The activity was quantified by measuring the decrease in absorbance at 340 nm by oxidation of NADPH in the presence of excess glutathione reductase (GR) for 5 min. Results are expressed as milliunits (mU) of GPx activity per milligram of protein (mU GPx/mg protein).

- Oxidized (GSSG) and reduced (GSH) glutathione concentrations

Both GSSG and GSH were measured following a fluorometric assay^49^. This method is based on the ability of glutathione (both GSSG and GSH) to react with o-phthalaldehyde (OPT) at pH 12 and pH 8, respectively, forming a fluorescent compound. Peritoneal leukocytes (10^6^ cells/mL) were resuspended in phosphate buffer (pH 8, 50 mM, 0.1M EDTA). Samples were then sonicated, and 5 μL of HClO_4_ (60%) was added and centrifuged for 20 min at 3200 g. 10 μL of the supernatants were dispensed into dark-bottomed 96-well plates (one for GSSG and one for GSH). For GSSG quantification, 5 μL of N-ethylmaleimide (NEM, 0.04M) was added to the samples to prevent oxidation of existing GSH and incubated for 30 min. Subsequently, 190 μL of NaOH and 20 μL of OPT were added to each well, and fluorescence was measured at 420 nm. In the case of GSH, 10 μL of the supernatants were added to the opaque plates, followed by adding 190 μL of 50 mM phosphate buffer at pH 8 and 20 μL of OPT, and fluorescence was measured at 420 nm. Results are expressed as nanomoles (nmol) of GSSG or GSH per milligram of protein (nmol GSSG/mg protein; nmol GSH/mg protein). In addition, the GSSG/GSH ratio was calculated.

- Concentration of thiobarbituric acid reactive substances (TBARs)

Quantification of TBARs was performed using the commercial “Lipid Peroxidation Assay Kit” (Biovision). Peritoneal leukocytes (10^6^ cells/mL) were resuspended in 300 μL of lysis buffer (containing butylated hydroxytoluene; BHT), sonicated, and centrifuged at 13,000 g for 10 min. Supernatants were collected, mixed with 600 μL of thiobarbituric acid (TBA), and incubated in a 95 °C water bath for 60 min. Then, 300 μL of butanol were added and centrifuged at 1700 g for 15 min, allowing extraction of the organic phase. 200 μL of this were collected and transferred to a 96-well plate and absorbance was measured at 532 nm. Results are expressed as nanomoles (nmol) of TBARs per milligram protein (nmol TBARs/mg protein).

### Protein quantification

Protein assessment was carried out on the same supernatants collected from the analysis of the different redox parameters. Protein quantification was performed by the bicinchoninic acid (BCA) method, using the BCA kit, which is based on the reduction of Cu^2+^, generating Cu^+^ ions that bind to BCA and form a colored compound that absorbs light at 562 nm. The results were expressed in milligrams of protein per milliliter (mg protein/mL).

### Statistical analysis

To develop the mathematical model for estimating BA in mice, a multiple linear regression analysis was performed using the SPSS 27.0.1.0 statistical program. For the Immunity Clock model, CA of the mice was introduced as an dependent variable and as predictor variables: phagocytic index and efficacy, macrophage and lymphocyte chemotaxis, Natural Killer activity, basal and concanavalin A and lipopolysaccharide stimulated lymphoproliferation, and concanavalin A and lipopolysaccharide stimulation percentages. For the Redox Clock model, CA of the mice was introduced as an dependent variable and as predictor variables glutathione peroxidase activity, glutathione reductase activity, the concentration of oxidized and reduced glutathione, GSSG/GSH ratio, and concentration of TBARs. Both models were generated through multiple linear regression following the “step forward” methodology, which first selects the variable considered most explanatory for the prediction of the dependent variable and then selects and adds the rest of the variables to the model one by one if they satisfy the condition of being statistically significant once they are incorporated into the model with *p *< 0.05. The models were tested for normality of the residuals, constant variability of the residuals (homoscedasticity), and the independence of the residuals was tested to verify the Gauss-Markov hypothesis through the corresponding graphical and analytical analysis.

Subsequently, the models were validated by calculating Pearson’s correlation coefficient between CA and BA and BA and lifespan in different sets of mice by using GraphPad Prism 8.4.1.

Finally, the differences between groups subjected to various lifestyle interventions were studied by performing a comparison of means (Student’s T-value for independent samples), considering a two-sided *p* < 0.05 as statistically significant.

## Data Availability

Data will be available upon request to Irene Martínez de Toda (imtcabeza@ucm.es) or Judith Félix (jufelix@ucm.es).
